# Completeness and Interpretability of Inpatient Pharmacist Documentation for Patients With Physician-Documented Asthma: A Retrospective Audit

**DOI:** 10.7759/cureus.115180

**Published:** 2026-08-25

**Authors:** Paweesuda Samsri, Pichaya Vorachak, Akaraphong Thaweephat, Kridsadadanudej Wongwejwiwat

**Affiliations:** 1 Department of Pharmacy, Sisaket Hospital, Sisaket, THA; 2 Faculty of Pharmacy, Siam University, Bangkok, THA

**Keywords:** asthma, clinical documentation, hospital pharmacy, inhaler technique, medication adherence, pharmacist documentation, quality improvement

## Abstract

Background: Routine inpatient pharmacist records can support continuity of care and service evaluation, but their usefulness depends on whether documented values are complete and clinically interpretable and whether the documentation distinguishes domains that were assessed and found negative from those that were not assessed or not documented. The objectives of this study were to describe medication documentation and asthma-management issues recorded by pharmacists for hospitalized patients with physician-documented asthma and to assess the structural completeness and interpretability of selected structured and free-text documentation fields.

Materials and methods: This retrospective documentation audit included 32 pharmacist-documented admission records from Sisaket Hospital, Thailand, from October 2024 through September 2025. To avoid duplicate observations from the same patient, the earliest eligible record for each hospital number was retained, resulting in 27 patients. Structured medication, comorbidity, and prior-exacerbation fields were audited for explicit values and missingness. Free-text notes were coded with an operational codebook by a primary coder and one of two alternating second coders, with disagreements resolved by consensus. Descriptive statistics and binomial 95% confidence intervals (CIs) were reported.

Results: The mean age was 61.19 ± 15.50 years, and 21 of 27 patients (77.78%) were female. All audited medication fields contained explicit binary responses, all comorbidity fields contained explicit text, and the prior-exacerbation field contained a numeric value for all patients. Pre-admission inhaled corticosteroid/long-acting beta2-agonist and long-acting muscarinic antagonist therapy were recorded in 19 of 27 (70.37%) and nine of 27 (33.33%) records, respectively. Both were positive in separate pre-admission fields in eight of 27 records (29.63%), although the explicit triple-therapy field was negative in every record. Pharmacist notes documented trigger- or exposure-related wording in 16 of 27 records (59.26%; 95% CI, 38.80%-77.61%), inhaler technique issues in 11 of 27 (40.74%; 95% CI, 22.39%-61.20%), and poor adherence wording in nine of 27 (33.33%; 95% CI, 16.52%-53.96%). One stand-alone “No” entry was unclassifiable because its original prompt was unavailable.

Conclusions: The selected records contained clinically relevant asthma-management wording, but the documentation structure limited the reproducibility of interpretation. The findings are documentation frequencies rather than hospital prevalence estimates, and do not establish the effectiveness of pharmacist care. Three-state assessment fields, separated exposure categories, and explicit active-regimen fields are needed before routine documentation can reliably support service evaluation or outcome research.

## Introduction

Asthma is a heterogeneous chronic respiratory disease that can require emergency treatment and hospitalization when control deteriorates. Inpatient review should address diagnostic certainty, recent instability, comorbidities, inhaled treatment, adherence, inhaler technique, and avoidable exposures [1-4]. The continuity and evaluability of this care depend not only on what clinicians assess but also on what the record can unambiguously represent.

Inhaled therapy is particularly documentation-sensitive. Clinically important inhaler errors are associated with poorer asthma outcomes, while adherence may be influenced by beliefs, regimen complexity, device burden, adverse effects, cost, and practical barriers [5-10]. A medication field that is positive for a drug class does not by itself establish current use, correct technique, or consistent adherence.

Pharmacists may contribute to medication reconciliation, medication review, inhaler assessment, adherence support, discharge counseling, and transitions of care [11-16]. However, evidence from structured pharmacist interventions should not be extrapolated to routine records when assessment status, intervention intensity, recommendation acceptance, issue resolution, and follow-up are incompletely captured. In this setting, the documentation itself becomes an important quality attribute of pharmacy practice.

Thai studies have demonstrated continuing clinical and economic burdens associated with asthma [17-19]. Interpretation of hospitalized asthma records may also be complicated by diagnostic heterogeneity, particularly in older adults in whom chronic obstructive pulmonary disease, asthma-chronic obstructive pulmonary disease overlap, and other causes of wheeze may coexist [1,2,20]. Despite these challenges, the interpretability of routine inpatient pharmacist documentation has received limited attention.

The objectives of this retrospective documentation audit were to (i) describe medication documentation and asthma management issues explicitly recorded by pharmacists for hospitalized patients with physician-documented asthma and (ii) assess the structural completeness and interpretability of selected structured and free-text documentation fields. The audit evaluated what was documented and how reliably the documentation could be interpreted; it was not designed to estimate hospital-wide prevalence, determine whether undocumented clinical domains had been assessed, evaluate clinical outcomes, or assess the effectiveness of pharmacist care.

## Materials and methods

Study design, setting, and ethics

This retrospective documentation audit used routinely collected pharmacist documentation and hospital records from Sisaket Hospital, a provincial hospital in northeastern Thailand. Eligible records were dated from October 1, 2024, through September 30, 2025. The Human Research Ethics Committee of Sisaket Hospital, Ministry of Public Health, Thailand, approved the study on July 14, 2026 (SSKHREC No. 067/2569; COA No. 040/2569). The requirement for informed consent was waived because the study used retrospective, de-identified records and involved no direct participant contact. No formal sample size calculation was performed because all available records that met the operational criteria during the study period were included.

Data source and patient selection

The source dataset contained 32 admission records with pharmacist documentation, hospital numbers, dates, demographic variables, structured medication and comorbidity fields, a numeric prior-exacerbation field, and a free-text issue field. The dataset was selected based on the presence of pharmacist documentation rather than on hospital-wide case identification. Consequently, the total number of asthma admissions and the proportion receiving pharmacist documentation were unknown.

To avoid duplicate observations from the same patient, records were ordered chronologically, and the first eligible record observed for each hospital number was retained. This process yielded 27 unique patients; later records from the same hospital number were excluded.

Operational case definition

Eligibility required the term “Asthma” in the diagnosis field and a pharmacist-documented date within the study period. The dataset did not contain International Classification of Diseases, 10th Revision codes, primary-diagnosis status, spirometry, structured adjudication of asthma-chronic obstructive pulmonary disease overlap, or systematic exclusion of alternative causes of wheeze. The audit therefore concerns records labeled as asthma by a physician and does not claim a validated cohort of acute asthma exacerbations.

Audit of structured variables

Medication fields were stored as explicit yes/no responses. All reviewed pre-admission and in-hospital medication fields were audited for missingness; blank values were not converted to “No.” The comorbidity field was reviewed for the presence of explicit text, including “No” when no comorbidity was recorded. The prior-exacerbation field was a pre-existing numeric source field labeled as the number of exacerbations during the 12 months before admission and was audited for the presence of a numeric value. The available retrospective dataset did not include an operational definition of an exacerbation or the method used to ascertain this count; therefore, the variable was treated as a source-recorded value rather than as a validated clinical endpoint. Variable-specific denominators were used. One invalid date sequence, in which the recorded discharge date preceded the admission date, was excluded only from the length-of-stay calculation.

Medication categories could overlap. The source-labeled “combination bronchodilator” field did not identify active ingredients, formulation, timing, or confirmation of active home use. Positive inhaled corticosteroid/long-acting beta2-agonist and long-acting muscarinic antagonist responses in separate fields were not combined to define confirmed triple therapy because the source fields did not establish simultaneous active use. These paired positive fields were therefore reported only as a documentation pattern, while the explicit triple-therapy field was retained and reported separately for comparison.

Coding of pharmacist free-text documentation

The free-text field was not a validated drug-related problem instrument and did not establish whether each clinical domain had been assessed. It was analyzed as pharmacist-recorded asthma management wording using four operational categories: trigger- or exposure-related wording, inhaler technique issue, poor adherence wording, and ambiguous or unclassifiable text. More than one category could be assigned to the same record. The complete operational codebook is provided in the Appendix.

The primary coder coded all 27 records. Each record then received a separate second review by one of two additional investigators who alternated this role. Differences were resolved through discussion and consensus. Only consensus classifications were retained in the analytic dataset; therefore, valid pre-consensus percentage agreement and Cohen's kappa could not be reconstructed.

Trigger- or exposure-related coding required explicit wording about smoking, smoke, dust, or another exposure; structured smoking status alone was insufficient. A recurring source phrase combined smoking, smoke, and dust and could not be separated into specific exposure types. Technique coding required an explicit error, such as poor actuation-inhalation coordination or inadequate breath holding. Adherence coding required explicit wording such as non-compliance, missed doses, or irregular use. The absence of wording was classified as not documented; it could not distinguish a domain that was not assessed from one that was assessed and found negative. A stand-alone “No” was classified as ambiguous because the original prompt was unavailable and was excluded from issue-frequency numerators.

Statistical analysis

Analysis was descriptive and patient-level. Continuous variables were summarized using the mean and standard deviation, and the median and interquartile range. Categorical variables were summarized as number and percentage with variable-specific denominators. Binomial 95% confidence intervals (CIs) were calculated for the three main free-text categories. No hypothesis tests, regression models, readmission analyses, or P values were used. Values were recalculated from the cleaned patient-level dataset and checked against the source dataset. Statistical analyses were performed using R version 4.5 (R Foundation for Statistical Computing, Vienna, Austria).

## Results

Record selection and patient characteristics

The source dataset contained 32 pharmacist-documented admission records. Retaining the first observed eligible record for each hospital number yielded 27 unique patients. The mean age was 61.19 ± 15.50 years, and 21 of 27 patients (77.78%) were female. Of the 27 patients, never-smoking status was recorded for 23 (85.19%). Hypertension was recorded in nine (33.33%), diabetes mellitus in five (18.52%), dyslipidemia in three (11.11%), and chronic kidney disease in two (7.41%). The median source-recorded number of exacerbations during the preceding 12 months was 0.00 (interquartile range (IQR), 0.00-1.00). One invalid discharge-date sequence was excluded from the length-of-stay calculation, leaving 26 patients for that variable (Table 1).

**Table 1:** Patient-level characteristics at the first observed eligible record (N=27) The prior-exacerbation field was labeled as the 12 months before admission and contained a numeric value for all 27 patients. Length of stay used n=26 because one record had a discharge date earlier than its admission date.

Characteristic	Value
Age (years), mean ± SD	61.19 ± 15.50
Age (years), median (IQR)	61.00 (52.00-69.50)
Female sex, n (%)	21 (77.78)
Never smoker, n (%)	23 (85.19)
Hypertension, n (%)	9 (33.33)
Diabetes mellitus, n (%)	5 (18.52)
Dyslipidemia, n (%)	3 (11.11)
Chronic kidney disease, n (%)	2 (7.41)
Prior exacerbations during preceding 12 months, median (IQR)	0.00 (0.00-1.00)
Length of stay (days), median (IQR)	2.00 (1.00-3.75); n=26

Medication documentation

All audited medication fields contained explicit yes/no values for the 27 first observed eligible records. Pre-admission inhaled corticosteroid/long-acting beta2-agonist therapy was recorded in 19 patients (70.37%), long-acting muscarinic antagonist therapy in nine (33.33%), and the source-labeled combination bronchodilator in 16 (59.26%). Inhaled corticosteroid/long-acting beta2-agonist and long-acting muscarinic antagonist fields were both positive in eight pre-admission records (29.63%). In contrast, the explicit pre-admission triple-therapy field was negative in all 27 records (100%).

During the index hospitalization, inhaled corticosteroid/long-acting beta2-agonist and long-acting muscarinic antagonist fields were both positive in seven of the 27 records (25.93%). In contrast, the explicit in-hospital triple-therapy field was negative in every record. These paired-field observations indicate a documentation-classification inconsistency and do not confirm simultaneous active use (Table 2).

**Table 2:** Medication documentation at the first observed eligible record Note: All medication fields contained explicit yes/no responses; no blank values were treated as “No.” Categories may overlap. Separate positive inhaled corticosteroid/long-acting beta2-agonist and long-acting muscarinic antagonist fields may represent open triple therapy, but simultaneous active use could not be confirmed.

Medication Field	Frequency (Percentage)
Pre-admission inhaled corticosteroid/long-acting beta2-agonist	19 (70.37)
Pre-admission long-acting muscarinic antagonist	9 (33.33)
Pre-admission combination bronchodilator, as recorded	16 (59.26)
Pre-admission inhaled corticosteroid	5 (18.52)
Pre-admission short-acting beta2-agonist	7 (25.93)
Pre-admission explicit triple therapy	0 (0.00)
Inhaled corticosteroid/long-acting beta2-agonist and long-acting muscarinic antagonist recorded in separate pre-admission fields	8 (29.63)
Intravenous corticosteroid during index admission	25 (92.59)
Nebulized bronchodilator during index admission	27 (100.00)
Nebulized budesonide during index admission	12 (44.44)
In-hospital inhaled corticosteroid/long-acting beta2-agonist	22 (81.48)
In-hospital long-acting muscarinic antagonist	8 (29.63)
Inhaled corticosteroid/long-acting beta2-agonist and long-acting muscarinic antagonist recorded in separate in-hospital fields	7 (25.93)

Pharmacist-recorded asthma-management wording

After duplicate review and consensus coding, trigger- or exposure-related wording was documented in 16 records (59.26%; 95% CI, 38.80%-77.61%), an inhaler technique issue in 11 (40.74%; 95% CI, 22.39%-61.20%), and poor adherence wording in nine (33.33%; 95% CI, 16.52%-53.96%) (Table 3). These values are documentation frequencies within the selected records, not prevalence estimates. The trigger- or exposure-related category could not be divided reliably into active smoking, passive smoke, dust, occupational exposure, or other environmental factors because the recurring source wording bundled several terms. One record contained only “No”; it was classified as ambiguous or unclassifiable and excluded from the three issue-frequency numerators.

**Table 3:** Pharmacist-recorded asthma-management wording (N=27) Note: Each record was reviewed by the primary coder and one of two alternating second coders; final values are consensus classifications. More than one category could be assigned. Absence of wording was treated as not documented, not assessed-negative. One stand-alone “No” was ambiguous and excluded from the numerators.

Documentation Category	Frequency	Percentage	95% Confidence Interval
Trigger- or exposure-related wording documented	16	59.26	38.80%-77.61%
Inhaler technique issue documented	11	40.74	22.39%-61.20%
Poor adherence wording documented	9	33.33	16.52%-53.96%

Structural documentation completeness and interpretability

The audit distinguished structural completeness from clinical interpretability. Structured medication fields were structurally complete because every audited field contained an explicit binary response; however, this did not establish active regimen status, timing, formulation, or simultaneous use of multiple medications. The comorbidity and 12-month prior-exacerbation fields were structurally complete in the 27 selected records because each contained an explicit source value. Structural completeness should not be interpreted as evidence that a clinical domain was comprehensively assessed or that the recorded information was clinically validated. In contrast, the free-text field did not distinguish a domain that was assessed and found negative from one that was not assessed or not documented. Table 4 summarizes the principal documentation-quality observations.

**Table 4:** Structural documentation completeness and interpretability audit Note: Structural completeness indicates the presence of an explicit source value and does not establish clinical validity, comprehensive clinical assessment, active treatment status, or standardized assessment.

Field or Observation	Records	Audit Interpretation
All pre-admission and in-hospital medication fields	27/27	Explicit binary responses; no blank source values, but active regimen status, timing, and formulation were not established.
Comorbidity field	27/27	Explicit text in every record, including “NO” when no comorbidity was recorded.
Prior-exacerbation count	27/27	Explicit numeric count labeled as the 12 months before admission.
Ambiguous free-text entry “No”	1/27	Prompt unavailable; classified as ambiguous and excluded from issue-frequency numerators.
Separate positive pre-admission inhaled corticosteroid/long-acting beta2-agonist and long-acting muscarinic antagonist fields	8/27	May represent open triple therapy, but simultaneous active use could not be confirmed.
Explicit pre-admission triple-therapy field	0/27	Inconsistent with separate medication fields in eight records; interpreted as a classification problem.

## Discussion

This retrospective audit identified clinically relevant wording on exposures, inhaler technique, and adherence in selected inpatient pharmacists' records, along with structural limitations that constrained interpretation. Its contribution is not an estimate of the prevalence of these problems among all hospitalized patients with asthma. Rather, it demonstrates what the available documentation can and cannot support when routine records are reused for service evaluation.

The documented inhaler technique and adherence issues are consistent with established asthma-care priorities and with evidence linking critical inhaler errors and non-adherence to poorer outcomes [5-10]. Nevertheless, an explicit phrase confirms only that a problem was recorded. When a record contains no wording for a domain, the audit cannot distinguish a negative assessment from a lack of assessment or incomplete documentation. A reproducible form should therefore provide at least three states for each domain: problem present, assessed and absent, and not assessed or not documented.

The exposure category illustrates a second semantic problem. The recurring source phrase combined smoking, smoke, and dust, preventing separation of active smoking, second-hand smoke, dust, occupational exposure, biomass exposure, and other triggers. This bundled field may be practical for rapid narrative entry, but it is unsuitable for domain-specific surveillance or targeted intervention evaluation. Separate structured exposure types, accompanied by an optional free-text field, would preserve both analyzability and clinical detail.

Medication fields were structurally complete in the narrow sense that every audited field contained an explicit yes/no value, but structural completeness did not guarantee clinical interpretability. Broad labels did not establish active ingredients, dosage form, timing, or whether a medicine represented an active home regimen. Separate positive inhaled corticosteroid/long-acting beta2-agonist and long-acting muscarinic antagonist fields could be compatible with open triple therapy, but they did not confirm simultaneous active use. Their inconsistency with a negative explicit triple-therapy field is therefore best interpreted as a documentation-classification or data-model problem rather than a clinical finding. Future documentation should capture the medicine, device, dose, frequency, start/stop status, and whether therapies were active concurrently.

The mean age above 60 years and the frequent positive findings for long-acting muscarinic antagonists and combination bronchodilators also underscore the possibility of diagnostic heterogeneity. Without diagnosis codes, primary-diagnosis status, spirometry, or structured overlap adjudication, the study could not verify acute asthma exacerbation in every record. Accurate classification is important because asthma admissions and readmissions can be misclassified when alternative or overlapping respiratory diagnoses are not explicitly addressed [1,2,20].

Pharmacist-led interventions can improve selected medication-use processes, inhaler technique, adherence, and transitions of care [11-16]. This audit, however, did not measure the number or intensity of pharmacist activities, recommendations made, prescriber acceptance, issue resolution, discharge medication changes, or follow-up. The observed documentation should therefore not be interpreted as evidence of a standardized intervention or of the effectiveness of pharmacist care.

Strengths and limitations

A strength of the study was the deliberate restriction of claims to explicit source documentation. Medication missingness was audited directly, free-text categories were defined in an operational codebook, and every record received a second review before consensus.

At the same time, this study has several limitations. It was based on a small, single-center, asthma-specific, documentation-selected sample, and the hospital-wide asthma denominator was unknown. Diagnostic specificity was limited because diagnosis codes, primary-diagnosis status, spirometry, and structured adjudication of asthma-chronic obstructive pulmonary disease overlap were unavailable. The retrospective dataset also lacked an operational definition or ascertainment rule for the source-recorded 12-month exacerbation count. The free-text categories were not validated, and the absence of wording could not distinguish a domain that was not assessed from one that was assessed and found negative. Coder-specific decisions before consensus were not preserved, medication-field semantics were ambiguous, and reliable discharge and follow-up outcomes were unavailable. Accordingly, the reported documentation frequencies may not generalize to other hospitals, diseases, or documentation systems. These limitations preclude hospital-wide prevalence estimation, causal inference, and outcome evaluation.

Recommendations

The audit nevertheless provides a practical redesign agenda. A prospective electronic form should identify each assessed domain, the finding, the pharmacist's action, whether a recommendation was accepted, whether the issue was resolved, the discharge regimen, and the follow-up plan. Hospital-wide case identification should retain diagnosis codes, objective respiratory evidence when available, and explicit distinction among asthma, chronic obstructive pulmonary disease, and overlap. A subsequent re-audit could then determine whether the redesigned documentation improves completeness, interpretability, and continuity of care.

## Conclusions

Among 27 documentation-selected hospitalized patients with physician-documented asthma, pharmacist notes contained trigger- or exposure-related wording in 16 records, inhaler technique issues in 11, and poor adherence wording in nine. The audit identified structural documentation problems that limited interpretability, particularly the inability of free-text documentation to distinguish assessed-negative findings from unassessed or undocumented domains, bundled exposure wording, and medication fields that did not reliably establish active regimen status and timing. Routine documentation may be more suitable for quality evaluation if each clinical domain uses explicit three-state assessment fields, exposure types are separated, and medication fields clearly identify the active regimen and timing. Prospective implementation and re-audit of redesigned documentation could determine whether these changes improve documentation completeness and interpretability.

## Appendices

Purpose and scope

This codebook was used to classify explicit wording in the pharmacist free-text issue field from the first observed eligible record retained for each patient. The codes describe what was documented and do not establish confirmed clinical prevalence or whether every domain was assessed.

General coding rules

The coding unit was one retained patient record. More than one category could be assigned to the same record. Codes were based solely on explicit free-text wording; no findings were inferred from structured smoking status, medication fields, or the absence of wording. Absence of wording was treated as not documented, not as evidence that a domain had been assessed and found negative.

**Table 5:** Operational codebook for pharmacist-recorded asthma-management wording Note: Multiple categories can be assigned to a single record. The absence of wording was treated as not documented, not as an assessed negative finding.

Category	Operational definition	Inclusion criteria and examples	Exclusion rules	Ambiguous cases and final handling
Trigger- or exposure-related wording documented	Explicit free-text wording describing exposure to a possible asthma trigger or environmental risk.	Assign when the note explicitly mentions smoking, smoke, dust, or another stated exposure. Other eligible examples, if present, include occupational, biomass, pet, or allergen exposure.	Do not assign from structured smoking status alone. Do not infer a specific exposure type from broad or bundled wording.	The recurrent source phrase combined smoking, smoke, and dust. Such records were assigned only to the broad trigger- or exposure-related category and were not separated into active smoking, second-hand smoke, dust, or another exposure.
Inhaler technique issue documented	Explicit free-text identification of an error or inadequacy in inhaler use.	Assign for an identified technique error such as poor actuation-inhalation coordination, failure to hold the breath, or breath holding documented as only three seconds.	Do not assign for counseling, demonstration, or review unless an actual technique error is documented. Absence of wording is not an assessed-negative result.	When adequacy was unclear, the record was reviewed during consensus discussion and coded only when an explicit technique problem could be supported.
Poor adherence wording documented	Explicit wording describing medication use that was inconsistent with the prescribed treatment plan.	Assign for wording such as “non-compliance,” missed doses, irregular use, or an equivalent explicit description of patient medication-taking behavior.	Do not assign for medicine unavailability, a regimen change, poor disease control, or another circumstance unless the wording explicitly describes inconsistent patient use.	Wording that could reflect access, prescribing, or regimen issues rather than patient use was coded only when consensus review supported an explicit adherence meaning.
Ambiguous or unclassifiable free-text entry	Text that could not be linked to a defined domain because the original prompt or clinical context was unavailable.	Assign to entries such as a stand-alone “No” when no prompt or surrounding context is available.	Do not interpret the entry as absence of all asthma-management issues or as evidence that all domains were assessed and found negative.	Report separately and exclude from the trigger/exposure, inhaler technique, and poor adherence frequency numerators.

Duplicate coding and consensus procedure

The primary coder coded all 27 retained records. Each record received a separate second review by one of two additional investigators, who alternated this role. Differences were resolved through discussion and consensus. Only the consensus classifications were retained in the analytic dataset; therefore, valid pre-consensus percentage agreement and Cohen’s kappa could not be reconstructed retrospectively.

Numerator Rule

The stand-alone “No” entry was classified as ambiguous or unclassifiable and excluded from the numerators for the three main documentation categories. Denominators remained the 27 retained patient records, as reported in this study.
